# Dismissal informs the priorities of endometriosis patients in New Zealand

**DOI:** 10.3389/fmed.2023.1185769

**Published:** 2023-06-01

**Authors:** Katherine Ellis, Deborah Munro, Rachael Wood

**Affiliations:** ^1^Department of Chemical and Process Engineering, University of Canterbury, Christchurch, New Zealand; ^2^Department of Mechanical Engineering, University of Canterbury, Christchurch, New Zealand; ^3^The Biomolecular Interaction Centre, University of Canterbury, Christchurch, New Zealand

**Keywords:** endometriosis, dismissal, hysteria, patient experiences, priorities, New Zealand, chronic pain, perspectives

## Abstract

**Introduction:**

Endometriosis is a common condition with average delays to diagnosis in New Zealand of almost 9 years.

**Methods:**

In total, 50 endometriosis patients participated in anonymous, asynchronous, online group discussions about their priorities, and their experiences with the development of symptoms, seeking a diagnosis, and receiving appropriate treatment.

**Results:**

Higher subsidy of care was the top change endometriosis patients wanted, followed by more research funding. When asked to choose whether research should be focused on improving diagnosis or improving treatment methods, the results were evenly split. Within this cohort, patients highlighted that they did not know the difference between normal menstrual discomfort and pathological endometriotic pain. If, upon seeking help, medical practitioners classified their symptoms as “normal,” these dismissals could instill doubt in patients, which made it more difficult for them to continue to seek a diagnosis and effective treatments. Patients who did not express dismissal had a significantly shorter delay from symptom onset to diagnosis of 4.6 ± 3.4 years vs. 9.0 ± 5.2 years.

**Conclusion:**

Doubt is a frequent experience for endometriosis patients in New Zealand, which was reinforced by some medical practitioners who were dismissive of their pain and thus prolonged the patient's delay to diagnosis.

## 1. Introduction

Endometriosis is a prevalent disease, with 10–15% of women (and people assigned female at birth) (1–3) expected to have endometrial-like tissue outside of the uterus (4, 5). Patients experience an array of symptoms, including but not limited to chronic pelvic pain, infertility (6), menstrual pain and distress (dysmenorrhea), pain with urination and defecation (dysuria and dyschezia), and pain with sex (dyspareunia) (7), all of which were expressed in this cohort.

This wide variety of symptoms is one aspect of why endometriosis is difficult to diagnose and treat (8).

Doctors misbelieving patients about their symptoms or downplaying the severity of symptoms can result in endometriosis patients having to “doctor shop.” In a 2004 North American study, 47% of women with endometriosis had seen at least five doctors before getting an endometriosis diagnosis or referral (9). In a New Zealand-based survey study from 2022, the average number of general practitioners (GPs) whom endometriosis patients had seen was 4.8 (10). This may be partially explained by the results of a 2021 French study, where 25% of GPs did not think they knew enough about endometriosis for their clinical practice (11).

Endometriosis is a disease with significant knowledge gaps. Historical underfunding contributes to issues with the limited range of ways it can be diagnosed and treated (12). These limitations relate to how GPs can assess the disease. Since three of the four stages of endometriosis are rarely identifiable on scans (13), the only other method available to doctors to make definitive diagnoses is surgery, the “gold standard” for endometriosis diagnosis (14).

Adding to the issue associated with obtaining an endometriosis diagnosis is an ongoing trend in the treatment of females with pain. It has been shown that women are more likely to have a chronic pain condition and are at increased risk of chronic pain (15), but have immensely different experiences of accessing treatment for their pain than men (16). Women dominate reports of “medically unexplained” diagnoses, which doctors are prone to disbelieve as root causes of pain (17), and are more likely to have their pain described as “psychogenic” (16). In a study assessing the perspective of healthcare practitioners of male and female patients in pain, there was a pattern of men in pain being described as stoic, tolerant or denying their pain, autonomous, avoiding healthcare, and not talking about their pain and its relationship to their wellbeing (18). Meanwhile, women were described as more sensitive to pain, more willing to report pain, more used to internal pain, “hysterical, emotional, complaining, not wanting to get better, malingerers,” and pain fabricators (18).

These interpretations translate into the care of female patients in pain. In a US study, with similar mean pain scores, male patients waited an average of 49 min in the emergency room when they presented with abdominal pain, whereas female patients waited for 65 min (19). Similarly, a 2022 US study found that when presenting with chest pain women were significantly less likely to be viewed as emergent, and waited 11 min longer to be seen by a provider than men (20). The physical appearance of women, but not men, is also important in the assessment of their pain. Women who look too “attractive” and therefore too healthy are less believable as sick, while women who look unwell are considered unreliable narrators (21). On average, women are prescribed lower quantities and less effective pain relief (17) and more antidepressants than men (22). Furthermore, a tendency has been shown by some providers to have lower goals for pain relief for chronic pain patients, such as endometriosis patients, compared to patients experiencing acute pain (23).

Previous studies that have assessed research priorities for endometriosis have included a mixture of patients, advocates, researchers, and medical professionals (24, 25). One of these studies (an online survey about research priorities conducted in the United Kingdom and Ireland) found that 70.3% of the 1225 participants had diagnoses of endometriosis, and 20.2% were healthcare practitioners (24). The 2017 meeting of the Global Consortium of Investigators also addressed research priorities, which resulted in the publication of 106 endometriosis research goals (25). The purpose of setting these research goals was to improve patient outcomes while acknowledging the complexity of tackling endometriosis, particularly with the limited availability of funding for research globally. Three of the new recommended research goals considered patient perspectives. One of these goals was to determine “patient views on the most pressing topics in endometriosis research and clinical priorities” (25). The purpose of the current study is to address this research goal with a New Zealand cohort, with an emphasis on assessing costs, barriers, and priorities for the future.

Endometriosis patient perspectives are regularly ignored and undervalued by clinicians (26), and it is important that these patient perspectives are not minimized or marginalized, and instead, are accessible to both academics and clinicians.

Within this article, aspects of a study conducted with endometriosis patients in New Zealand in 2022 are presented. The first article about this study concerned themes of symptom intensity, diagnostic shortcomings, imposter syndrome, life-changing diagnosis, and perceptions of treatment efficacies. Key findings of that article included that the predominant emotion at diagnosis among the cohort was relief (86%), only 25% of users of the combined oral contraceptive pill found it effective for their pain, and the average delay to confirmed diagnosis was 8.6 years (27). The present article discusses the role of insurance and private care, the need for more subsidized care, the desire for more research funding, the barrier to patient knowledge, and the power of the practitioner.

## 2. Materials and methods

The methods used to collect and analyze the data, the description of the patient cohort, and the limitations of this study were described in detail in Ellis et al. (27) and are briefly described below.

### 2.1. Recruitment

In February and March 2022, snowball social media advertising was used to recruit participants. Interested participants contacted the first author and were provided with an information sheet and a consent form. The selection criteria applied were as follows: participants had to be over the age of 18, reside in New Zealand, and have a diagnosis of endometriosis that was either confirmed by surgery (“confirmed diagnosis,” 84.0% of participants) or suspected by their GP or OBGYN (“working diagnosis,” 16.0% of participants). No further selection criteria were applied, and all participants who expressed their interest, fulfilled the criteria, and filled in the consent form were eligible to participate. In total, 81 people expressed interest, 59 completed the consent form, and of these 59, 50 participated in the research and 9 did not complete the questionnaire. Details of the participants' diagnosis status, age, and disease stage are given in Supplementary Table 1.

### 2.2. Data collection

The questionnaire comprised 50 questions, of which 23 had text answers and 27 were polls (Supplementary material). The online discussions were asynchronous and ran for 72 h. Each participant had a unique link to the discussion and could log in asynchronously and anonymously to answer questions, read responses, and write replies. To reduce “groupthink” (28), participants could only see the anonymized responses of other participants once they had submitted their own answers.

### 2.3. Demographics

**Figure 1:**
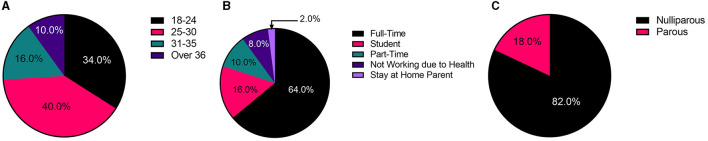
Summary of participant demographics. **(A)** Age distribution. **(B)** Working status. **(C)** Parity.

The participants were aged between 18 and 48 years and were a mixture of full and part-time workers, students, stay-at-home mothers, and individuals who were not working, and there was a mixture of people who were nulliparous (have not had children) and parous (had one or more pregnancies) (Figure 1).

### 2.4. COVID-19 and online research

The surge of COVID-19 during the period of this research meant that the online environment was a requirement, as has been reported by other researchers (29). The online research environment has other benefits, including increased data volume, reduced cost, increased accessibility for participants, and allows recruitment from a larger geographical area (30).

### 2.5. Data analysis

Quantitative answers were collated in GraphPad (version 9), and qualitative answers were collated into a single transcript. The transcript was read in full twice before coding with an inductive, iterative thematic approach. Coded quotes were transferred into a spreadsheet where themes and sub-themes were iteratively organized. The key themes and sub-themes identified in this study are summarized in Supplementary Table 2.

### 2.6. Ethical approval

This research was approved by the University of Canterbury Human Research Ethics Committee (Ref: HREC 2022/03).

### 2.7. Comparison of priorities calculation

The endometriosis patients in this study selected and ranked their top three selections for what they wanted for the future out of six options. The six changes selected by endometriosis patients include the following: more social awareness, more research funding, more social acceptance, more education and information, more subsidized care, and more support groups. Choices were assigned points (three points for their first choice, two for their second, and one for their third), which were totaled for each change, and then expressed as a percentage of the total points available (6 points per participant, 50 participants, and 300 total points available).

### 2.8. Statistical analysis

Statistical analysis of the data was completed in GraphPad (version 9). In all figures, (^*^) indicates a *p*-value of < 0.05, (^**^) indicates a *p*-value of < 0.01, and (^***^) indicates a *p*-value of < 0.001. The null hypothesis in all cases was that the means of all levels were equal. Shapiro–Wilk tests were conducted to determine whether data were normally distributed. Since data were non-normally distributed, unpaired Mann–Whitney *t*-tests were used to compare means.

## 3. Results and discussion

### 3.1. The need for subsidized care

The endometriosis patients in this study selected and ranked their top three of six changes (out of six options) they most want to see for patients in the future. The highest-rated selection was more subsidized care at 35.8%, followed by more research funding (23.7%) and more education and readily available information (20.4%).

In regards to subsidized care, patient experiences indicated the costs of treatment were unacceptable and made it nearly impossible to obtain in a timely manner without paying privately for treatment or using medical insurance, as highlighted by one participant (25–30, Confirmed, Nulliparous): “Diagnosis and treatment should not be withheld due to your financial position.”

#### 3.1.1. Differences in public and private healthcare

**Figure 2:**
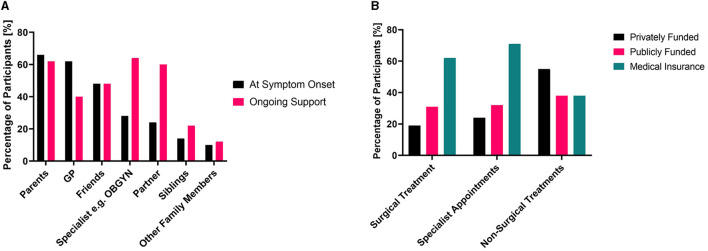
**(A)** Comparison of the people who endometriosis patients relied on for support at the time of symptom onset and for ongoing support. **(B)** The method used to pay for consultations and treatments, multiple selections possible for both.

The graphs in Figure 2 show the key people that endometriosis patients turned to when they first started experiencing symptoms vs. who they rely on now for their ongoing support. While 62% of participants initially turned to their GP for treatment, only 40% relied on their GP for ongoing support. Meanwhile, while only 28% of participants initially turned to a specialist, such as an OBGYN, for care at symptom onset, this proportion increased to 64% for ongoing support. The trend of decreased support from GPs and increased specialist support is likely the result of two key factors. First, it was common that after becoming disheartened with their treatment, or with the time required to wait for referral or treatment, patients made the choice to pay for private medical insurance or healthcare. Second, with increasing age and with an increasingly extensive medical history of experiencing endometriosis symptoms and failure of frontline treatments, patients were finally able to obtain a referral from their GP for specialist care. Patients in this cohort expressed that wait times for treatment in the public health system (which is available for free to all New Zealanders) were too long and led to the need to either pay privately or live with debilitating pain:

“Waitlist for referrals was incredibly hard to deal with. 6 months per referral and did not get to Women's Health for years. Currently waiting up to 6 months for my pain clinic referral, as unfortunately I now have chronic neuropathic pain.” (18–24, Confirmed, Nulliparous)

“If it was easier to be seen within the public system I feel like I would be suffering much less! I know the system is overwhelmed and the pandemic has not helped the situation, but for those of us who cannot afford private healthcare, waiting months or years for support is just not good enough.” (18–24, Confirmed, Nulliparous)

“Wait times are so long and providers are so inadequate in the public system that people are forced to consider how to pay for private insurance and alternatives.” (25–30, Working, Nulliparous)

Delays in treatment not only can worsen symptoms (31) but can force patients to take out medical insurance or pay out-of-pocket for private care. The extensive wait times in public healthcare mean a portion of patients is systematically excluded from obtaining the “gold standard” laparoscopic confirmation of endometriosis because of their socioeconomic status. Only 31% of patients with confirmed diagnoses were able to have their surgery funded through the public system, while other patients utilized medical insurance (62%) and paid privately (19%) for part or all of their surgical costs (Figure 2). This trend was also true for specialist appointments—with 71% of patients utilizing medical insurance and 24% paying privately for part or all of the specialist costs. Only 32% of patients relied upon the public system for payment for specialist appointments. This indicates many patients rely on private means of paying for either treatment or insurance to cover their care. Many patients shared that they viewed costs as a substantial barrier to accessing treatment:

“If someone requires treatment to be able to live a normal life, the cost of it should never have to be a barrier.” (18–24, Confirmed, Nulliparous)

“Cost is such a huge barrier. It is one thing to know you have it (and awareness is important) but if you cannot afford to do anything about it, that can feel so disheartening and overwhelming.” (25–30, Working, Nulliparous)

“No woman should have to choose between paying for necessary surgery or medicines vs. being able to afford to survive and pay her bills.” (25–30, Confirmed, Nulliparous)

There were vast differences between the experiences of patients with private healthcare and those in the public system. Many patients reported that as soon as they were paying privately for treatment (either out of pocket or using medical insurance), they were treated better by their practitioner and received working diagnoses far more rapidly. When discussing their experiences with their specialists, in comparison to their GPs, patients highlighted that their OBGYNs tended to be better at listening to their symptom histories, taking them seriously, being supportive and sympathetic, and offering more resources:

“I knew there was something wrong and have been fighting to get a diagnosis. It has been near impossible to get any type of diagnosis except through private care… I found support was behind a paid wall. Only private were able to help/assist me, which I had to pay for or get insurance for. General GPs were no support.” (18–24, Working, Parous)

“5 years later at 23 I was diagnosed with endometriosis after going privately and being seen by a gynecologist (what a relief!) there were many happy tears shed after my laparoscopy. Endometriosis was growing in my bladder, bowel and ovaries.” (25–30, Confirmed, Nulliparous)

#### 3.1.2. The effect of medical insurance on outcomes

It was evident within this study cohort that having medical insurance had a dramatic impact on the experiences of endometriosis patients. As one patient (18–24, Working, Parous) explained: “Because endometriosis is a pre-existing condition I could not get my health insurance to pay for my private surgery scheduled a few months down the track. I, therefore, went onto the public waiting list which can take years.” Medical insurance allowed better access to specialist appointments, physiotherapy, prescriptions, and vitally, surgery. The benefits of having medical insurance before symptom onset, or before seeking help for endometriosis symptoms, were frequently discussed, as a pre-existing condition of endometriosis symptoms limited medical insurance options.

One patient (18–24, Confirmed, Nulliparous) recounted their experience of being told they did not have enough symptoms to be referred for further endometriosis care and surgery—unless they had medical insurance. Since the patient did have medical insurance, they were referred. When the specialist gave the patient a pamphlet, they noticed that despite their GP's dismissiveness, they had every single symptom listed.

Patients were highly aware that having medical insurance changed the outcome of their experience substantially. When asked what advice they would give an endometriosis patient at the start of their experience, one patient (18–24, Confirmed, Nulliparous) said “SPEAK UP AND DO NOT STOP UNTIL YOU ARE IN SURGERY. Not every professional knows what they are doing. Professionals get things wrong. And before you ever tell a doctor about symptoms… Get medical insurance! I pay a hefty price for mine now and I would rather go without a phone, car, food than not have it.” Other experiences with medical insurance included:

“I claimed almost $100,000 with my health insurance provider, very few people would be able to financially manage.” (31–35, Confirmed, Nulliparous)

“Because of my family's medical insurance, other than the premiums, it did not cost us a cent. Therefore thankfully [cost] was not a barrier for my treatment.” (18–24, Confirmed, Nulliparous)

“Having insurance cover my appointments and surgery has been the best thing ever—I would have had to ration appointments and surgeries otherwise.” (31–35, Confirmed, Nulliparous)

### 3.2. The need for more research funding

The second-highest ranked change (23.7%) that endometriosis patients wanted to see was an increase in the research funding allocated to endometriosis. Internationally, endometriosis is an underfunded research endeavor, relative to both condition prevalence and the economic burden to patients and nations (12). However, this does not mean that no research into endometriosis occurs, or conversely that the research that occurs is always beneficial. When asked what they wanted to see, some endometriosis patients highlighted a concern that resonated with them:

“There needs to be more research funding into endometriosis itself, how it impacts the lives of women and what treatments are actually effective... I am so angered by the amount of funding put toward research based on things like how endometriosis affects the sex lives of men. Men do not have any idea, and if a cure or even just more effective treatments are found then it would not be an issue for men anyway. Such a waste of funding in my opinion.” (18–24, Confirmed, Nulliparous)

“If you are putting your time into our disease, put it into making sure you are going to help improve our lives, not my boyfriend's sex life!” (18–24, Working, Nulliparous)

The type of research these participants are referring to fails to address critical knowledge gaps. There have been multiple studies into the effect of endometriosis symptoms on partners (32), with some intentionally focusing on their male partners (33–35). The emphasis of these studies is often the influence of patient endometriosis on their partner's sex life. Part of the issue of such studies is they utilize researcher time, funding, and effort without closing any of the numerous, vital knowledge gaps about endometriosis and how it affects those who live with the disease. Patients within our study's cohort highlighted that the lack of research funding directly translates into fewer options for the diagnosis and treatment of their condition.

Patients in our cohort were asked the binary choice response question of whether they would rather have research conducted into improving the diagnosis of endometriosis, or the treatment of endometriosis. The results showed that 50% wanted treatment of endometriosis to be improved, while 50% wanted the diagnosis of endometriosis improved. Many patients highlighted the question was difficult to answer as both desperately needed improvement. The result of this line of questioning is clear—patients want and need both the capacity to obtain a timely diagnosis of endometriosis and have more treatment options for the chronic and debilitating disease. If one of these aspects of endometriosis care was functioning successfully, it would be expected to see an apparent selection by patients.

Improvements to diagnosis and treatment cannot exist in a vacuum and must be implementable both in clinical practice and in the lives of endometriosis patients. Patient priorities are vital in the development of solutions that can reduce the onus of the disease on patients, reduce the delay to diagnosis, and improve quality of life. It is also evident from these results that patients want to see research into their condition, and specifically, improving their condition, conducted. The patients of this study highlighted three key aspects that ideal future solutions should be as follows: (1) non-invasive, (2) non-hormonal, and (3) readily accessible irrespective of the patient's personal financial situation.

### 3.3. Improving patient knowledge

According to the findings of this study, understanding what endometriosis is and how it manifests is an integral part of the delay in diagnosis. At symptom onset, the majority of participants knew very little (34%) or had never heard of endometriosis (52%). In contrast, only 4% considered themselves very knowledgeable (2%) or well-informed (2%) (Table 1). It was common among participants to describe their initial belief that the pain and suffering they experienced were within “normal” parameters. This was often endorsed by educators and medical professionals. As an example, one patient (18–24, Confirmed, Nulliparous) recalled an endometriosis health class where the teacher described the pain as so severe that it would cause sufferers to vomit and faint. Therefore, the teacher claimed, “I'm sure none of you have it.” Even though the participant's dysmenorrhea resulted in tears, difficulty breathing and absences from work and school, due to this teacher's statement, they were sure that their condition was not severe enough to be endometriosis and feared talking about their considerable pain would seem exaggerated and they would be seen as an attention seeker.

**Table 1:** Patient level of understanding of endometriosis at symptom onset and source patients first heard about endometriosis.

**Variable**	**Percentage of patients**
**Level of understanding at symptom onset**	***n*** = **50**
I had never heard about endometriosis	52%
I knew very little about endometriosis	34%
I knew a bit about endometriosis	10%
I was well-informed about endometriosis	2%
I was very knowledgeable about endometriosis	2%
**Source patients first learnt about endometriosis**	***n*** = **50**
From online research about my symptoms	30%
My GP or OBGYN told me about it	22%
From a friend	14%
School education program	14%
From a family member	10%
From a fiction novel	4%
From another endometriosis patient	2%
From a teacher	2%
Flyer, advertisement or public service announcement	2%

Patients within this cohort were clear that increasing knowledge about endometriosis would benefit patients, both by increasing their ability to recognize their symptoms as endometriosis earlier and by ensuring the people around them are better able to understand their condition:

“Having more education around the condition would be amazing as it was not something that was discussed during high school.” (18–24, Confirmed, Nulliparous)

“There needs to be more material for younger girls to reference from school and there needs to be education in all high schools about this disease. Many suffer for unreasonable lengths of time and are told to get on with it and how normal the pain is. It's not normal!” (31–35, Confirmed, Parous)

“More education for all students on a normal period, [both] girls and boys so it is not a taboo subject.” (25–30, Confirmed, Nulliparous)

#### 3.3.1. The lack of knowledge about endometriosis

The data displayed in Table 1 highlight the historically low level of education and awareness of endometriosis in New Zealand. The delivery of an education program in schools has increased adolescent awareness and is associated with a shift to earlier presentation to specialized health services seeking endometriosis treatment (36). During a meeting for the Society for Women's Health Research (Washington, DC), increased education and disease awareness were highlighted as critical areas for improving the diagnosis, treatment, and care of endometriosis patients (37). Patients within this cohort highlighted that without effective education they were frequently unaware their experiences were abnormal and indicative of endometriosis:

“I thought it [really heavy bleeding, migraines, cramps, sore back and legs] was just what all girls went through on their periods.” (18–24, Working, Nulliparous)

“I went undiagnosed for so long because I did not know enough to think I could have it, so I ignored my symptoms.” (18-24, Confirmed, Nulliparous)

When patients were asked where they had first heard about endometriosis, the most common source was through their own online searches about their symptoms (30%). A total of 24% learned about endometriosis from a friend (14%) or a family member (10%) which exceeded hearing about endometriosis from a medical practitioner (22%). In this study cohort, only 14% of participants learned about endometriosis through an educational program at school. Since the incidence of endometriosis symptoms often relates to menstruation and the age at which menarche (first menstrual period) occurs in developed countries, which tends to be between the ages of 10 and 16 (38), it would be beneficial to increase the prevalence of education aimed at middle school and high school students.

New Zealand was the first country in the world to have a Menstrual Health and Endometriosis program in secondary schools when the program was started in 1997 (39). Learning about endometriosis from a school program was revolutionary for one participant (25–30, Confirmed, Nulliparous), who said: “I was incredibly lucky… during [the] talk I ticked off every single symptom… I then felt comfortable going back to my GP and demanding investigation instead of just various contraceptive pills and ‘deal with it'.”

#### 3.3.2. Belief that endometriosis is rare

Endometriosis affects 1 in 10 women (and people assigned female at birth) in New Zealand (40), with up to 130,000 patients currently experiencing the condition. Importantly, in New Zealand, endometriosis is almost as prevalent in women as breast cancer (breast cancer affects one in nine women in New Zealand) (41), as prevalent as diabetes in women (42) and 4.3 times as prevalent as rheumatoid arthritis in women (43). Interestingly, although they were aware of endometriosis, a few participants were under the impression that endometriosis was rare. The belief in rarity or knowledge of family or acquaintances with endometriosis, which could have been beneficial to patients by increasing their awareness of the disease, could instead become a hindrance to diagnosis, as shown in the statement by one participant (25–30, Confirmed, Nulliparous): “I was told endometriosis was very rare, and when a close friend was diagnosed in high school, I thought the possibility of us both having it would be zero. I spent years talking to my (always male) GP before finally getting a referral.”

#### 3.3.3. Resources

For endometriosis patients to benefit from existing sources of information about endometriosis, they need to know what endometriosis is, where to look for information about endometriosis, realize they may have endometriosis, and then be able to easily interpret the information provided. Even patients who became aware of endometriosis often struggled to locate resources, as highlighted by one participant (25–30, Confirmed, Nulliparous) who shared: “I did not have any resources when I first started having symptoms, so at that point, anything would have been great.” There was a common sentiment among this study's cohort that resources, such as easily digestible pamphlets were lacking or unavailable for them. This may explain the reliance upon internet resources.

Social media resources, such as Facebook and Instagram, were consistently among the highest-rated sources by this cohort, with 32% highlighting their use for support and/or resources. Participants praised the social media accounts of both organizations and influencers. The patients within the study shared that they appreciated the insight from people who had experienced endometriosis personally and the sense of community associated with groups and followings, as explained by one participant (18–24, Confirmed, Nulliparous): “Instagram helped me follow other peoples' endometriosis diagnosis journey and realize the severity of the illness. I also found comfort in reading through subreddits about endometriosis.” This aligns with an Australian study that found that 76.0% of endometriosis patients reported positive psychological, social, and cognitive outcomes from their use of social media (44). The participants in our study highlighted that these groups could also be an effective way for patients to find out about doctors who may be more supportive of their efforts to obtain diagnosis and treatment, and doctors to avoid.

### 3.4. The influence of poor medical practitioner advice

Within this study, participants were misdiagnosed with cystic ovaries, anxiety, recurrent urinary tract infections (UTIs), “just period cramps,” food allergies, pelvic inflammatory disease, depression, premenstrual dysphoric disorder, “just a stress response,” and psychosomatic pain. In a 2012 study in Austria and Germany, 74.3% of endometriosis patients had experienced a misdiagnosis (45). However, one participant (36+, Confirmed, Nulliparous) raised a poignant point about misdiagnoses: [I had no misdiagnoses] “To misdiagnose, you have to listen.” Not only were patients frequently misdiagnosed with other conditions, but patients were gaslit into believing abnormal symptoms are a normal part of the female experience:

“My whole menstruating life I suffered from all the symptoms of endometriosis, but was gaslit by GPs that my pain levels and periods were normal—or could not be helped.” (25–30, Confirmed, Nulliparous)

“The doctors in my hometown were not forthcoming in the diagnosis in my teens because ‘this is normal for girls your age.”' (25–30, Confirmed, Nulliparous)

Endometriosis patients within this cohort frequently explained they had to obtain information about endometriosis for themselves to understand their experiences and felt let down by their doctors. As one participant put it: “[I wish I had] known more about [endometriosis] and stood up for myself. [I wish] for the doctors who did not listen to me to be held accountable for the harm they caused.” Within the endometriosis cohort, there were two key pieces of misinformation endometriosis patients were frequently told by their doctors:

1. You should get pregnant because:
  (a) It will make your symptoms go away or,
  (b) Now is the only chance you will have to become pregnant
2. You do not have:
  (a) the correct, or
  (b) enough symptoms for a diagnosis of endometriosis

#### 3.4.1. Pregnancy is not a cure for endometriosis

It is stated in the European clinical guidelines for endometriosis that the suggestion to “get pregnant” should never be made to endometriosis patients (46). The key study that uses clinical data to make the claim that pregnancy will improve endometriosis is from 1991 and included a total of 41 participants, with the 16 people in the pregnant group only in the first trimester of their pregnancy (47). In a 2018 review of evidence from 1966 to 2017, the authors concluded that there was no evidence that pregnancy would generally reduce the size or number of endometriotic lesions (48). Furthermore, a systematic review in 2016 found that pregnancy can be associated with complications, with a presently unquantified incidence due to a lack of sufficiently large epidemiological studies (49).

Despite the minimal evidence to support the claim that pregnancy is a cure for endometriosis, many of the patients within the cohort had doctors suggest they should get pregnant to cure their symptoms, including patients who indicated that they did not want children. Participants also explained that their pregnancies had caused further pain. As many as half of endometriosis patients suffer from fertility problems which may relate to issues such as chronic inflammation and anatomical distortions (50, 51). The insistence by medical practitioners to some of the patients in this study that they should urgently become pregnant led to feelings of surprise, upset, or a sense of being misled among the patients:

“One doctor even suggested the best plan was to have a baby! I was only early 20's and so not ready for a baby so was taken aback when this was suggested to me.” (36+, Confirmed, Parous)

“[I felt] scared because I worried this would be something I had to tackle forever. Scared at 21 I would never have kids. Told I had my best chance if I tried before 25. 4 years left to be a kid. This changed my whole life experience: instead of taking a gap year, I worked through university, got a job, and bought a house, all to prepare for my best chance at having a child. I was robbed of freedom because of inept doctors who gaslit me.” (18–24, Confirmed, Nulliparous)

“[My gynecologist] suggested I think about having children sooner as it could help [reduce pain]. I was 19 at the time! By the time I had [my child] and the pain I had during pregnancy— just stretching etc.—I had a feeling it would continue.” (31–35, Confirmed, Parous)

#### 3.4.2. Dismissal of endometriosis symptoms

Multiple endometriosis patients were told they could not be diagnosed with endometriosis because they lacked enough symptoms or because the symptoms they had, regardless of how debilitating, were insufficient for a diagnosis. Lacking mid-cycle pain and bleeding, or heavy, painful, irregular periods were enough for their GPs to rule out endometriosis. Meanwhile, other participants were told by their doctors that “severe period pain, heavy bleeding and length of bleeding was normal” (25–30, Confirmed, Nulliparous) and “painful periods alone were not anything to be too concerned about” (18–24, Confirmed, Nulliparous).

Throughout this study cohort, some participants that shared pain with periods were frequently dismissed as normal and they were made to feel “crazy” upon the dismissal of their symptoms, and the blame for their symptoms was put on them. While up to 50% of patients with deep infiltrating endometriosis will experience lower UTIs due to endometriotic lesion invasion or surgical trauma (52), one endometriosis patient in this cohort (with stage IV endometriosis) was told her constant UTIs were just the result of her continuing to wear synthetic underwear and having poor hygiene. Similarly, food allergies were assigned the blame for abdominal pain and bloating. One participant (25–30, Confirmed, Nulliparous) explained that “for years I believed and felt terrible that my suffering these symptoms was because of my own actions.” Multiple participants received referrals for psychiatric support, as their experiences were blamed on anxiety, depression, and stress:

“I was told to try a meditation app and maybe yoga as stretching would help with my pain. She basically told me it was all in my head and ‘if you let go of your anxious thoughts most of these things you are feeling will just go away.”' (25–30, Confirmed, Nulliparous)

“It took a lot to convince doctors I was not faking or being overly dramatic. One even suggested I looked up the symptoms and [faked my symptoms].” (36+, Confirmed, Parous)

#### 3.4.3. The benefits of having a supportive doctor

Conversely to the pattern of medical practitioner dismissal harming patients, causing further delays to diagnosis and the development of doubt, other patients found that their experience of endometriosis care improved by their medical practitioner. One patient (31–35, Confirmed, Nulliparous) highlighted that their best advice was to: “Find a GP or specialist who (funny [that] this is like a lucky result, rather than what everyone experiences normally) acknowledges you may be one of the 1 out 10 who have endometriosis and makes it obvious that their focus is to learn about your symptoms and how they might be relieved.” Patients who were believed by the first GP they visited about their explanation of their experience found they accessed diagnosis and treatment far more rapidly than patients who were dismissed during their initial doctor visits for endometriosis. Patients who had been dismissed as dramatic or malingerers, who later found a different GP who believed and supported them, found that when they were believed and supported it sped up their efforts to obtain a diagnosis:

“I had a very attentive GP [who] identified the symptoms and immediately referred me to a specialist.” (31–35, Confirmed, Nulliparous)

“The final GP I saw who referred me to a specialist (she will never know how much that changed my life), she was the first GP I ever saw who said I think you have [endometriosis].” (25–30, Confirmed, Nulliparous)

“I went through a number of GPs before I found my current one. She was sympathetic and did not dismiss me as a malingerer.” (36+, Confirmed, Nulliparous)

“After about 10 years of symptoms, a random GP I saw in Auckland suggested [endometriosis] and sent me to a specialist in Auckland, who promptly diagnosed me (within about 6 weeks of seeing him I had surgery).” (31–35, Confirmed, Nulliparous)

### 3.5. The impact of doubt on endometriosis patients

A pattern evident throughout the discussion in this cohort was that patients were not aware their level of pain was unusual, or if they did believe it was abnormal, they were led to believe that they were being dramatic or overly sensitive. The majority of patients had never been educated in school or by medical professionals that their pain was not within the range considered non-pathogenic, which delayed them from seeking help to reduce their symptoms. Furthermore, endometriosis pain is complex and can impact regions seemingly unrelated to menstruation, as seen by reports of pain symptoms in areas such as the lower back, legs, and digestive tract in this cohort. This means many endometriosis patients, without the support of resources that highlight the variety of locations that can be affected by endometriosis-related pain and symptomology, did not connect the occurrence of these symptoms with the disease.

**Figure 3:**
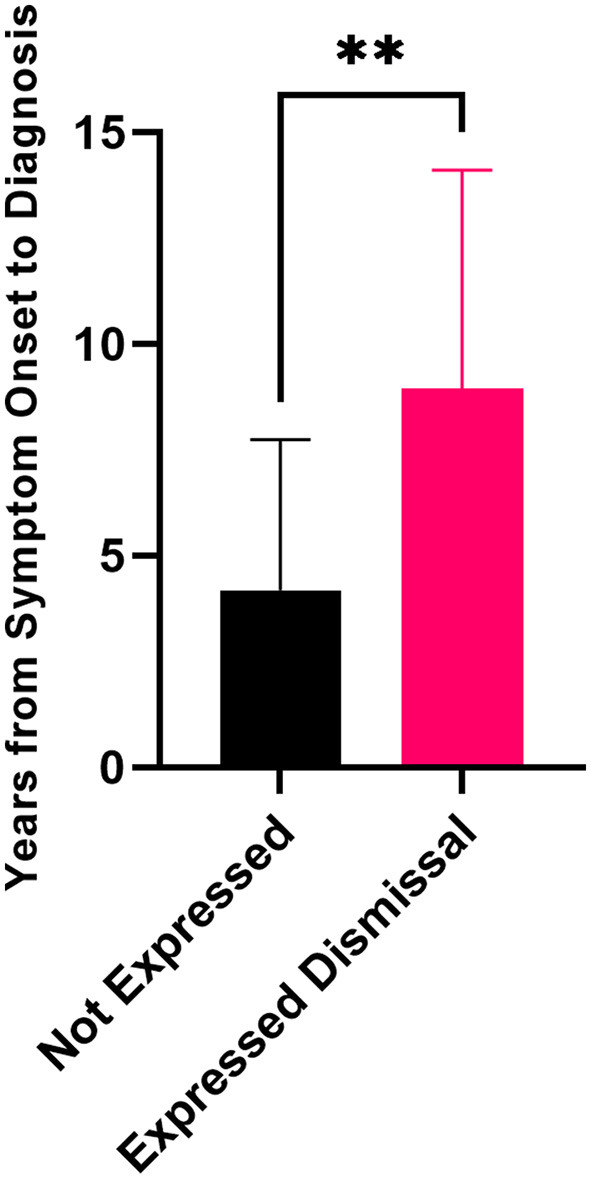
Delay to diagnosis for patients who expressed dismissal was a part of their journey to diagnosis (*n* = 36) compared to patients who did not express these sentiments (*n* = 11) (unpaired Mann–Whitney *t*-test, **p = 0.0017). Error bars represent standard deviation.

When the delay from symptom onset to diagnosis was compared, the patients who expressed doubt, dismissal, a lack of belief from medical practitioners, or a sense they were gaslit or not being taken seriously had statistically significantly longer delays to diagnosis compared to patients that did not express similar sentiments (Figure 3). Patients who expressed these concerns had average delays of 9.0 ± 5.2 years vs. 4.6 ± 3.4 years for the patients that did not.

#### 3.5.1. “I did not know my pain was not normal”

Endometriosis patients consistently shared they were unaware that their pain was pathogenic, and assumed it was within the normal range for people who menstruate. Many patients were told by family members, or even doctors, that their pain tolerance was just low, and there was no need for any actions to alleviate that pain. For some participants, this inaccurate reinforcement that their pain was normal, and it was instead their thoughts about the pain that was abnormal, left them with inherent doubts that they had endometriosis. For some patients, the effects of doubt lingered even after an endometriosis diagnosis were confirmed. The experience of one patient (25–30, Confirmed, Nulliparous) was that even after four surgeries had confirmed endometriosis, they still felt the “first 3 years of being told I was crazy have hugely informed my ability to trust myself and my body.” Similar accounts from other patients included:

“I did not understand that the pain I was in was NOT NORMAL. I spent so much time wondering if I was crazy or even just being weak because all uterus-having people get periods and I just need to suck it up and deal with it like everyone else does.” (31–35, Confirmed, Nulliparous)

“I just assumed I was making a big deal about nothing… who was to say that my suffering was any more than any other woman's” (36+, Confirmed, Parous)

#### 3.5.2. How gender informs the care of females in pain

One participant (36+, Confirmed, Nulliparous) highlighted an assumption they had faced from medical professionals that “women have a higher tolerance for pain, which is nonsense and used as an excuse for neglecting patients with conditions that are associated with women.” This assumption is not supported by research. Rather than having a higher pain tolerance, women have a higher pain sensitivity compared to men (15, 53, 54).

The perceived normality of pain for endometriosis patients often delayed their diagnoses and delegitimized their own sense of suffering. Some patients were told, or had it written on their medical records, that what they were experiencing was “hysteria”:

“I have had radiologists comment on my reports saying: “endometriosis unlikely due to birth control” and even “likely hysteria.” What is this the 1850′s?” (18–24, Confirmed, Nulliparous)

“[Endometriosis] definitely influenced how I thought about periods and being a female. I hated having my period, I thought I was disgusting and never really had a positive outlook on being a woman.” (31–35, Confirmed, Parous)

“I wish I were taken more seriously! At one point I was told it is just “female hysteria” I felt insane. The only time I got somewhere was when I was working with a female doctor, when dealing with a male doctor I was constantly told that everything was normal without more investigation. I just wish concerns were taken more seriously.” (18–24, Confirmed, Nulliparous)

#### 3.5.3. How clinicians view endometriosis patients

In a 2018 Australian study assessing the perspectives of endometriosis clinicians, the clinicians indicated that medicine was the authoritative source of knowledge for women and their bodies, and they viewed endometriosis patients for whom treatment had been unsuccessful or who disagreed with the clinician's perspective as “difficult” patients (26). “Difficult patients” they explained, would continually seek care following a lack of symptomatic relief from repeated treatment attempts. This frustrated the clinicians, who, in turn, identified fault and responsibility within the patient, and continually stated or implied the role of hysteria, or a psychosomatic source of continued symptoms (26).

Clinician referrals are a key barrier to effective treatment and confirmed diagnoses, as they not only prescribe medication but control public referrals to specialist care in New Zealand, so patients are required to submit to the perspective of their clinician. In this study's cohort, the weight of doubt was monumental and was often exacerbated by interactions with their clinicians. In the 2018 Australian study, the clinicians indicated that they were able to infer the accuracy of a patient's account of their experiences by assessing their behavior, and the application of their objective medical knowledge could allow them to determine what was “actually” happening within the patient's body (26). Clinicians disliked when patients placed their experiential knowledge of their condition over the expertise of the clinician. When the patients in this cohort were dismissed by the medical authority and told they knew less about their experience than their clinician, it created immense pressure, which either forced the patient to find a new clinician or to dismiss their experiences and go without help:

“But I will never forget a particular gastroenterologist who was convinced I was just stressed because he simply could not find a result. He questioned my own belief and while in his room I walked out, burst into tears and began to lose hope.” (25–30, Confirmed, Nulliparous)

“I am a healthcare professional and even my own diagnosis of stage 3 [endometriosis] was delayed by at least 7 years.” (25–30, Confirmed, Nulliparous)

“Completely understand the imposter syndrome, right up until the moment I woke up and they told me I had [endometriosis] I felt so anxious I was going to go through it all and find nothing and be at square one and labeled a drama queen.” (18–24, Confirmed, Nulliparous)

Trivialization and dismissal of endometriosis patients and their symptoms by medical practitioners have been reported before (55, 56), and in some cohorts was the worst part of their experience as well as a key cause in delaying diagnosis (57). Endometriosis pain involves a complex disease process where pain tolerance decreases with the duration of symptoms (58). Endometriosis is a condition where the two frontline approaches to treatment, birth control, and pain relief are often considered ineffective by patients (27), leading endometriosis patients to be perceived as “difficult patients” who refuse to get better (26), rather than as patients being given relief options that are not working for their bodies or their condition.

### 3.6. Recommendations

Confirmed endometriosis diagnosis has been highlighted before as a key step for patients experiencing endometriosis symptoms to feel legitimized as patients (27, 59); however, the delay to diagnosis from symptom onset remains high at 8.7 years (10), longer than delays reported in the USA (60), the Netherlands (61), and the UK (62), but within an accepted average global range of 4–11 years (63). In order to reduce the delay to diagnosis in New Zealand, and in other countries around the world, three key aspects must be prioritized for improvement (64), the very three highlighted as the priorities of this cohort. First, an investment must be made into increasing specialist capacity and availability in the public health system to effectively serve and support the large cohort of patients needing this care. As highlighted in this study, it should also be ensured that GPs in New Zealand have sufficient knowledge about endometriosis to ensure they are not unintentionally dismissing patients presenting with these symptoms, thereby prolonging their delay to diagnosis. Second, research funding, which has been historically low for endometriosis internationally (12, 25), must be increased to allow the discovery of readily accessible, non-invasive diagnostic, and treatment tools for all patients. Finally, awareness and education must be increasingly improved and enhanced. Patients, and the general public, must both be aware of the main symptoms of endometriosis and where they can access care and support for their condition. This may also reduce the dismissal and downplaying of the pain and symptoms that participants highlighted they had been exposed to in their relationships, families, schools, and workplaces.

### 3.7. Study limitations

Key limitations of this study have been discussed previously (27). In brief, since participation in this study was done by self-selection, this may have driven patients with more unsatisfactory experiences to engage in this study, potentially biasing the results toward negative experiences. The self-selection of patients to participate may have also resulted in an under-representation of patients with working diagnoses, who may not readily view themselves as “legitimate” endometriosis patients. Finally, despite 17.1% of the New Zealand population identifying as Māori (65), no patients in the study self-identified as Māori. This may cause the study to miss key barriers and priorities of Māori endometriosis patients. This gap is being assessed in a follow-up study.

## 4. Conclusion

Patients within this study cohort shared the vast differences between public and private healthcare. Patients who were able to access the private system reported greater understanding, belief, and support, and a decreased delay to access treatments, particularly surgery. Patients, either waiting or who had waited in the public system for consultations and treatments, reported that the wait times were nearly unbearable, forcing many to spend money on private care they could not readily afford.

The three key things patients wanted to change for endometriosis patients in the future were more subsidized care, increased research funding, and more readily accessible information. Patients highlighted that private care was the method many patients had to rely on to get effective treatment, but private costs and medical insurance could be prohibitively expensive for many, creating a socioeconomic barrier. Endometriosis treatment is vital for improved quality of life for endometriosis patients, and even with a public health system in New Zealand, continues to be behind a significant paywall for many patients.

In terms of increased research funding, endometriosis patients highlighted that research needed to be actively focused on improving their experiences of the disease, with reduced delays to diagnosis and improved treatment given equal weight by this cohort. The reports of low knowledge levels surrounding endometriosis at symptom onset indicate low accessibility of information about endometriosis, and patients consistently reported relying on online resources such as Google searches and social media. This trend was so pronounced that more patients had first heard of endometriosis from online searches (30%) than from their doctors (22%).

In this cohort, trivialization and dismissal were key causes of the doubt for endometriosis patients and influenced diagnostic delay by dissuading patients that they had endometriosis. Furthermore, not understanding their pain was abnormal left many patients to believe they were simply weak, which was often reinforced by adults and doctors telling them they had a low pain tolerance and needed to take more painkillers. The doubt endometriosis patients face, where some doctors tell them their symptoms are not pathological and thereby normalize their pain, could become instilled in the patients and make them unable to trust their bodies, even after an official diagnosis. This repeated dismissal of patients is actively almost doubling the average delay to diagnosis from 4.6 years to 9.0 years and is a vital area to address in New Zealand in the future.

## Data availability statement

The raw data supporting the conclusions of this article will be made available by the authors, without undue reservation.

## Ethics statement

The studies involving human participants were reviewed and approved by University of Canterbury Human Research Ethics Committee. The patients/participants provided their written informed consent to participate in this study.

## Author contributions

KE, DM, and RW contributed to the design of the questionnaire. KE recruited the participants, moderated the discussions, analyzed the results, and she is the primary author of this manuscript through conception and analysis. DM and RW have contributed equally to this manuscript through critical revision and editing. All authors contributed to this article and approved the submitted version.
