# Supplementary material for: Dismissal informs the priorities of endometriosis patients in New Zealand
Source: Front Med (Lausanne). 2023 Jun 1;10:1185769. doi: 10.3389/fmed.2023.1185769 (PMC10267318; doi:10.3389/fmed.2023.1185769)
Supplement: Supplementary file 1 [file Table_1.DOCX]

**Supplementary Table 1:** Characterization of Endometriosis Patient Study Participants.

| **Patient Number** | **Age** | **Diagnosis Type** | **Parity** | **Disease Stage (if known)** |
| --- | --- | --- | --- | --- |
| 1 | 18-24 | Working | Nulliparous | - |
| 2 | 18-24 | Confirmed | Nulliparous | Stage II |
| 3 | 18-24 | Confirmed | Nulliparous | Stage III |
| 4 | 36+ | Confirmed | Parous | - |
| 5 | 18-24 | Confirmed | Nulliparous | Stage I |
| 6 | 36+ | Confirmed | Nulliparous | - |
| 7 | 31-35 | Confirmed | Nulliparous | Stage III |
| 8 | 25-30 | Confirmed | Nulliparous | Stage IV |
| 9 | 18-24 | Confirmed | Nulliparous | Stage III |
| 10 | 18-24 | Confirmed | Nulliparous | Stage I |
| 11 | 18-24 | Confirmed | Nulliparous | Stage III |
| 12 | 25-30 | Confirmed | Nulliparous | Stage II |
| 13 | 25-30 | Confirmed | Nulliparous | Stage IV |
| 14 | 31-35 | Confirmed | Nulliparous | - |
| 15 | 36+ | Confirmed | Nulliparous | - |
| 16 | 25-30 | Confirmed | Nulliparous | - |
| 17 | 36-40 | Confirmed | Parous | - |
| 18 | 25-30 | Working | Nulliparous | - |
| 19 | 18-24 | Working | Parous | - |
| 20 | 25-30 | Working | Nulliparous | - |
| 21 | 18-24 | Working | Nulliparous | - |
| 22 | 25-30 | Confirmed | Nulliparous | - |
| 23 | 18-24 | Confirmed | Nulliparous | Stage III |
| 24 | 18-24 | Confirmed | Nulliparous | Stage II |
| 25 | 25-30 | Confirmed | Nulliparous | Stage III |
| 26 | 31-35 | Confirmed | Parous | Stage IV |
| 27 | 25-30 | Confirmed | Nulliparous | - |
| 28 | 18-24 | Confirmed | Nulliparous | - |
| 29 | 25-30 | Confirmed | Nulliparous | - |
| 30 | 25-30 | Confirmed | Nulliparous | Stage II |
| 31 | 31-35 | Confirmed | Parous | - |
| 32 | 25-30 | Confirmed | Nulliparous | Stage III |
| 33 | 25-30 | Confirmed | Nulliparous | Stage IV |
| 34 | 31-35 | Confirmed | Nulliparous | Stage II |
| 35 | 31-35 | Confirmed | Parous | Stage III |
| 36 | 25-30 | Confirmed | Nulliparous | Stage II |
| 37 | 25-30 | Confirmed | Nulliparous | Stage II |
| 38 | 25-30 | Confirmed | Nulliparous | - |
| 39 | 18-24 | Working | Nulliparous | - |
| 40 | 18-24 | Confirmed | Nulliparous | Stage III |
| 41 | 18-24 | Confirmed | Nulliparous | Stage IV |
| 42 | 25-30 | Confirmed | Nulliparous | Stage III |
| 43 | 31-35 | Confirmed | Parous | Stage IV |
| 44 | 25-30 | Working | Parous | - |
| 45 | 18-24 | Working | Nulliparous | - |
| 46 | 36+ | Confirmed | Parous | - |
| 47 | 25-30 | Confirmed | Nulliparous | Stage III |
| 48 | 31-35 | Confirmed | Nulliparous | Stage III |
| 49 | 18-24 | Confirmed | Nulliparous | Stage II |
| 50 | 25-30 | Confirmed | Parous | Stage IV |
