# Supplementary material for: Dismissal informs the priorities of endometriosis patients in New Zealand
Source: Front Med (Lausanne). 2023 Jun 1;10:1185769. doi: 10.3389/fmed.2023.1185769 (PMC10267318; doi:10.3389/fmed.2023.1185769)
Supplement: Supplementary file 2 [file Table_2.DOCX]

**Supplementary Table 2:** Thematic Structure of Findings

|  | **Theme** | **Sub-themes** |
| --- | --- | --- |
| 1 | Intensity of symptoms | - Debilitating experiences - Negative influence in the workplace - Negative influence on education |
| 2 | Diagnostic tool shortcomings | - Traumatizing trans-vaginal ultrasounds - Endometriosis missed in surgery |
| 3 | Imposter syndrome | - Patients with working diagnoses feel like imposters |
|  |  |  |
| 4 | Life-changing diagnosis confirmation | - Relief - Anger at doctors - Anger at the pain |
| 5 | Varied perceived treatment efficacies | - Dislike of the emphasis on hormonal treatment - Pain relief medication insufficient - Life-changing laparoscopies - Mixed perspectives of intrauterine devices - Transition to alternative non-prescription methods - Positive view of hysterectomy |
| 6 | The role of insurance and private care | - Experiences of long wait times for public system care - Transition from public system into private care |
| 7 | The need for more subsidised care | - Insurance viewed as necessary - Prohibitively expensive private care - Socioeconomic influence on capacity to access effective care |
| 8 | Desire for more research funding | - Angered at poor uses of funding - Faster diagnosis - Better treatments |
| 9 | Patient knowledge as a barrier | - Lack of knowledge at symptom onset about endometriosis - Misconceptions about endometriosis - Perception of their feelings of pain as weakness |
| 10 | Power of the practitioner | - Dismissal instils doubt - Endometriosis misconceptions - Support perceived to speed up diagnosis |
