# Supplementary material for: Dismissal informs the priorities of endometriosis patients in New Zealand
Source: Front Med (Lausanne). 2023 Jun 1;10:1185769. doi: 10.3389/fmed.2023.1185769 (PMC10267318; doi:10.3389/fmed.2023.1185769)
Supplement: Supplementary file 3 [file Data_Sheet_1.doc]

**Discussion Board Questions:**

1. ABOUT YOU
   1. **Introduction** To start off, would you please briefly introduce yourself (no names required) and tell us a wee bit about why you’re interested in participating in this discussion forum.
   2. **Current Situation with Endometriosis (Single Choice Poll)** Which of the following best describes where you currently are in your endometriosis journey?

Options:

Working diagnosis from my GP and/or OBGYN but not pursuing any particular treatment

Working diagnosis from my GP and/or OBGYN and pursuing non-surgical treatment (e.g. pain management or birth control)

Working diagnosis from my GP and/or OBGYN and waiting for surgery

Confirmed diagnosis and now post-surgery (or surgeries)

Other (Please Specify)

1. EXPERIENCE WITH ENDOMETRIOSIS
   1. **Onset Age** At what age did you first notice endometriosis symptoms?
   2. **Symptoms (Multi Choice Poll)** Which of the following symptoms have you experienced and believe are associated with your endometriosis?

Options

Chronic Pelvic Pain (for a period exceeding six months)

Dysmenorrhea (Period Pain)

Dyspareunia (Pain with Sex)

Dysuria (Pain with Urination)

Pain with Defecation

Ovarian Cysts

Mid-Cycle Pain

Metrorrhagia (Mid-Cycle Bleeding)

Diarrhea

Constipation

Infertility

Sub-Fertility

Pain with Ovulation

Fatigue

Nausea

Other (Please specify if comfortable)

- 1. **Level of Understanding (Single Choice Poll)** What level of understanding did you have about endometriosis when you started experiencing symptoms?

Options

4 – I was very knowledgeable about endometriosis

3 – I was well informed about endometriosis

2 – I knew a bit about endometriosis

1 – I knew very little about endometriosis

0 – I had never heard of endometriosis

- 1. **Effect of Understanding** Do you believe the amount of knowledge you had when you first experienced symptoms, influenced your early experiences of endometriosis?
  2. **Endometriosis Information (Single Choice Poll)** How did you FIRST hear about endometriosis?

Options

My GP or OBGYN told me about it

Education program at school e.g. The EndoME program

From a family member

From a friend

From online research about my symptoms

Flyer, advertisement, or public service announcement

Other (Please Specify)

- 1. **Early Resources** What endometriosis resources did you find most helpful when you first started having symptoms? If you did not have adequate resources, what would have been helpful?
  2. **Support (Multi Choice Poll)** When you first started having symptoms, which people did you turn to for support?

Options

GP

Specialist e.g. OBGYN

Parents

Siblings

Partner

Friends

Other family members

Teacher

School Health Staff e.g. Nurse

Workplace Health Staff e.g. Nurse

Endometriosis Support Group

Other (Please Specify)

- 1. **Ongoing Support (Multi Choice Poll)** Which people have provided your key ongoing support?

Options

GP

Specialist e.g. OBGYN

Parents

Siblings

Partner

Friends

Other family members

Other (Please Specify)

- 1. **Support Experience** Please tell us about your experience seeking support.

In your response please cover:

- - Any barriers to support
  - Any particularly supportive members of your network
  - Any issues you have faced

1. EXPERIENCE WITH DIAGNOSIS
   1. **Journey Story** Please tell us your endometriosis diagnosis story, including whatever details you are comfortable with sharing.

In your own words, please share your experiences with:

- - Developing symptoms
  - Any misdiagnoses
  - Getting a diagnosis
  - Time to diagnosis
  1. **Diagnostic Tools (Multi Choice Poll)** Which of the following tools were used to inform your diagnosis?

Options

Symptom history

Physical examination by a GP/OBGYN

Abdominal ultrasound

Transvaginal ultrasound

MRI

Laparoscopy

Specimen collection (e.g. blood test)

Other Diagnostic Test (Please Specify)

- 1. **Diagnostic Tools Experience** Please elaborate on each diagnostic method you have experienced. Please highlight anything that was particularly positive, or negative, about your experience.
  2. **Diagnosis Feelings (Multi Choice Poll)** Which of the following best describes how you felt when you first received your endometriosis diagnosis?

Options

Upset

Overwhelmed

Relieved

Scared

Angry

Confused

Other (Please Specify)

- 1. **Diagnosis Feelings** In your own words, please elaborate how you felt when you received your diagnosis. Have your feelings changed or evolved over time?
  2. **Time to Diagnosis** How long was it from when you first sought medical advice for endometriosis, to when you received your diagnosis? Were there any misdiagnoses along your journey?
  3. **Waitlist Times** How long have you waited for various diagnostic methods? E.g. Abdominal ultrasound, two months, laparoscopy, nine months, etc.
  4. **Things to Change** If there was one main thing you could change about your journey to your current diagnosis, what would it be?
  5. **Piece of Advice** What advice would you give someone at the start of their endometriosis journey regarding diagnosis?
  6. **Rate Experience (Single Choice Poll then text responses)** How would you rate your experience obtaining a diagnosis? What main reasons contributed to this rating of your experience?

Options

5 – Excellent

4 – Good

3 – Neutral

2 – Bad

1 – Terrible

- 1. **Surgery (Single Choice Poll)** Have you had surgery to treat your endometriosis?

Options

Yes

No (Skip to Question 4.1)

- 1. **Only for Surgery = Yes. Time to Surgery** After consulting with a specialist, how long did you have to wait for your surgical procedure?
  2. **Only for Surgery = Yes. Disease Stage (Multi Choice Poll)** What stage was your endometriosis at the time of surgery?

Options

Stage I

Stage II

Stage III

Stage IV

I do not know

1. EXPERIENCE WITH TREATMENT
   1. **Treatment Approach (Multi Choice Poll)** Which of the following treatment options have you experienced?

Options:

Pain relief medication e.g. Ibuprofen or Panadol

Neuropathic pain relief e.g. Amitriptyline

Oral Contraceptive Pill with Estrogen and Progesterone e.g. Levlen

Progesterone Only Pill e.g. Cerazette

Intrauterine Device (IUD) e.g. Mirena or Jaydess

Pelvic Floor Physiotherapy

Laparoscopic surgery

Other surgical procedure

Counselling

Fertility Treatments

Hysterectomy

` Hormone Replacement Therapy (HRT)

Exercise

Other (Please Specify)

- 1. **Effective Treatments (Multi Choice Poll and text response)** Which of the following treatment options have you both used AND found effective at treating your symptoms? For each successful treatment, please comment on how much time elapsed before you found the treatment effective.

Options:

Pain relief medication e.g. Ibuprofen or Panadol

Neuropathic pain relief e.g. Amitriptyline

Oral Contraceptive Pill with Estrogen and Progesterone e.g. Levlen

Progesterone Only Pill e.g. Cerazette

Intrauterine Device (IUD) e.g. Mirena or Jaydess

Pelvic Floor Physiotherapy

Laparoscopic surgery

Other surgical procedure

Counselling

Fertility Treatments

Hysterectomy

` Hormone Replacement Therapy (HRT)

Exercise

Other (Please Specify)

- 1. **Positive Experiences** Which endometriosis treatments have you had positive experiences with? Please explain.
  2. **Negative Experiences** Which treatments for endometriosis have you had negative experiences with? Please explain.
  3. **Advice** Based on your personal experiences with treatment, what treatments would you recommend someone at the start of their endometriosis journey discuss with their GP or OBGYN? Why?
  4. **Treatments** If you were to start your endometriosis journey again, please discuss:
- Which treatments would you like to start straight away?
- Which treatment would you like to skip altogether?
  1. **Wait Times (Single Choice Poll)** How would you rate the wait times you experienced for your **initial consult**? This refers to the first time you ever spoke with a medical professional (likely your GP or OBGYN) about your symptoms.

Options

5 – Excellent

4 – Good

3 – Neutral

2 – Bad

1 – Terrible

- 1. **Specialist Consults (Single Choice Poll)** Have you had specialist consults for your endometriosis?

Options

Yes

No

- 1. **Non-Surgical Treatment (Single Choice Poll)** Have you had non-surgical treatments for endometriosis? e.g. progesterone-only pills, neuropathic pain relief, pelvic floor physiotherapy…

Options

Yes

No

- 1. **(Only for Specialist = Yes) Wait Times (Single Choice Poll)** Overall, how would you rate the wait times you have experienced for **specialist consults**?

Options

5 – Excellent

4 – Good

3 – Neutral

2 – Bad

1 – Terrible

- 1. **(Only for Non-Surgical = Yes) Wait Times (Single Choice Poll)** Overall, how would you rate the wait times you have experienced for **non-surgical treatments** e.g. pelvic floor physiotherapy?

Options

5 – Excellent

4 – Good

3 – Neutral

2 – Bad

1 – Terrible

- 1. **(Only for Surgery = Yes) Wait Times (Single Choice Poll then text responses)** How would you rate the wait times you experienced for **surgical treatments**? After surgery was recommended, how long did you wait for surgical treatment?

Options

5 – Excellent

4 – Good

3 – Neutral

2 – Bad

1 – Terrible

- 1. **Availability (Single Choice Poll)** Overall, do you agree treatment for endometriosis has been readily available to you?

Options

5 – Strongly agree

4 – Agree

3 – Neutral

2 – Disagree

1 – Strongly Disagree

- 1. **(Only for Specialist = Yes) Cost (Single Choice Poll then text responses)** Overall, how do you rate the cost of **specialist** **consults**? Please explain your reasons for this rating. If you have seen more than one specialist, please expand upon that here.

Options

5 – Excellent

4 – Good

3 – Neutral

2 – Bad

1 – Terrible

- 1. **(Only for Non-Surgical = Yes) Cost (Single Choice Poll then text responses)** Overall, how do you rate the cost of **non-surgical treatments**? Please explain your reasons for this rating. Please elaborate on each non-surgical treatment and how you would rate it individually.

Options

5 – Excellent

4 – Good

3 – Neutral

2 – Bad

1 – Terrible

- 1. **(Only for Surgery = Yes) Cost (Single Choice Poll then text responses)** Overall, how do you rate the cost of **surgical treatments**? Please explain your reasons for this rating. If you have had more than one surgical treatment, please elaborate on that here.

Options

5 – Excellent

4 – Good

3 – Neutral

2 – Bad

1 – Terrible

- 1. **(Only for Specialist = Yes) Paying for Treatment (Multi Choice Poll)** How do you pay for your **specialist** **consults**?

Options

Privately

Publicly Funded

Medical Insurance

Other (Please Specify)

- 1. **(Only for Non-Surgical = Yes) Paying for Treatment (Multi Choice Poll)** How do you pay for your **non-surgical treatments**?

Options

Privately

Publicly Funded

Medical Insurance

Other (Please Specify)

- 1. **(Only for Surgery = Yes) Paying for Treatment (Multi Choice Poll)** How did you pay for your **surgical treatments**?

Options

Privately

Publicly Funded

Medical Insurance

Paid for as part of a research study

Other (Please Specify)

- 1. **Cost Elaboration** Please discuss how the costs of endometriosis treatments have influenced you.
  - Has the cost of treatments been a barrier for you and impacted the treatment options you could access?
  - Are the costs of treatments reasonable?
  - Have you needed assistance (e.g. from family) to pay for treatments?
  1. **Elaborate** Are there any other points you would like to raise about wait times, availability and costs of endometriosis treatment in New Zealand? If so, please elaborate.

1. FUTURE FOCUS
   1. **Changes (Ranking)** Please rank the top THREE most important changes that you would like to see in New Zealand from the following. Then, please explain the reasoning behind your choice of the most important factor.

Options

- - 1. More social awareness about endometriosis
    2. More research funding into endometriosis
    3. More social acceptance of endometriosis
    4. More education and readily available information about endometriosis
    5. More subsidised endometriosis care
    6. More support groups for endometriosis
  1. **Changes for Patients** What is the biggest change you would like to see regarding how endometriosis patients are viewed in New Zealand? This may be socially or medically. Why do you think this change should occur?
  2. **What Needs Research Focus (Single Choice Poll)** For future research, which of the following two areas is more important to you?

Options:

Improved Diagnosis of Endometriosis

Improved Treatment of Endometriosis

- 1. **Ideas** Do you have any suggestions for how the diagnosis and treatment of endometriosis can be improved?
  2. **Medical Device** The future: If a medical device was developed to diagnose or treat endometriosis, what would you want it to achieve? How could it have helped you in the past? How could it help you with your condition now?
